# Low-Cost 2D Index and Straightness Measurement System Based on a CMOS Image Sensor

**DOI:** 10.3390/s19245461

**Published:** 2019-12-11

**Authors:** Alain Küng, Benjamin A. Bircher, Felix Meli

**Affiliations:** Federal Institute of Metrology METAS, Lindenweg 50, 3003 Bern-Wabern, Switzerland; benjamin.bircher@metas.ch (B.A.B.); felix.meli@metas.ch (F.M.)

**Keywords:** 2D position sensor, 2D index sensor, straightness sensor, machine tool geometry correction

## Abstract

Accurate traceable measurement systems often use laser interferometers for position measurements in one or more dimensions. Since interferometers provide only incremental information, they are often combined with index sensors to provide a stable reference starting point. Straightness measurements are important for machine axis correction and for systems having several degrees of freedom. In this paper, we investigate the accuracy of an optical two-dimensional (2D) index sensor, which can also be used in a straightness measurement system, based on a fiber-coupled, collimated laser beam pointing onto an image sensor. Additionally, the sensor can directly determine a 2D position over a range of a few millimeters. The device is based on a simple and low-cost complementary metal–oxide–semiconductor (CMOS) image sensor chip and provides sub-micrometer accuracy. The system is an interesting alternative to standard techniques and can even be implemented on machines for real-time corrections. This paper presents the developed sensor properties for various applications and introduces a novel error separation method for straightness measurements.

## 1. Introduction

Machine tools and measuring machines need to be calibrated with high accuracy. For this purpose, all degrees of freedom (DoF) of all displacement stages need to be characterized. Most of the machines use line scales or interferometers to measure displacements of the moving components of stages, thereby relying on the accuracy and repeatability of the guidance to master lateral and angular displacements. Yaw and pitch can be measured using a three-beam interferometer system; however, roll and the two lateral displacements are more problematic to be measured with high accuracy. The standard procedure for straightness calibration is to use tactile probes running along a straight edge or an interferometric set-up using a Wollaston prism [1]. Since those two methods are quite cumbersome and expensive, using an optically collimated beam to establish a reference line pointing onto a position-sensitive detector (PSD) [2,3] or a four-quadrant diode (4QD) [4], with [5,6] or without a corner cube reflector [7], is also often suggested in the literature. However, the accuracy reported by all systems using this working principle hardly reach 1 µm [5]. This is insufficient for measuring machines with sub-micrometer accuracy.

Four-quadrant diode sensors are often used to measure the center of a laser beam with extreme accuracy. The position where the intensity on the four diodes is balanced repeats with nanometer precision; however, when departing from this center, their response is sensitive to the laser beam intensity, as well as the beam shape and size [8]. Implementations of four-quadrant diodes as straightness sensors, thus, require careful in situ calibration. Position-sensitive devices (PSDs) are inherently insensitive to the beam intensity, as well as its shape and size. They do not require calibration apart from non-linearity corrections, providing that the analog electronic also exhibits a good linearity. They are thus widely spread in optical position measuring systems. Nevertheless, the determination of the laser beam center is physically realized and cannot be influenced. Ambient light, thus, directly impacts the measurement. In addition, beam shape monitoring is not possible with such devices.

The system we present here is composed of a fiber-coupled, collimated laser beam pointing onto a complementary metal–oxide–semiconductor (CMOS) image sensor and offers sub-micron accuracy for optical straightness measurement. It is also low-cost, allowing implementation on all axes of our computed tomography system [9] for a dedicated real-time straightness measurement. As mentioned above, the idea of using a collimated beam pointing onto a detector, even a CMOS image sensor [10], to measure the straightness of a stage is often suggested, but a complete investigation of its accuracy has, to our knowledge, not yet been reported. This lack of understanding of the limiting effects prevented the implementation of the simple error separation method proposed here, which can reduce systematic errors to reach an accuracy down to a few tens of nanometers.

## 2. System Conception and Limitations

Digital image sensors, whether charge-coupled device (CCD) or CMOS type, with their pixels acting as a ruler to locate the optical beam center, provide many advantages. Due to their mass production, they may even be cheaper than PSDs or 4QDs. Since image sensors are manufactured with lithographic precision, one can expect a very high accuracy through sub-pixel interpolation [11]. Their digital interface renders them very convenient to use. Different beam center determination algorithms can be quickly tested. Subtraction of ambient light, linearization, or even more complex functions can be implemented. Nevertheless, the major drawback of digital image sensors versus other types of optical sensors is their power dissipation, which can lead to thermal drift of the chip position. Mastering the fluctuations of the power consumption of the chip over time is a prerequisite to reach a high accuracy.

As mentioned above, at fixed and very short distances from the optical source, optical positioning systems can exhibit a very high accuracy, sometimes even below a nanometer. At long distances from the optical source, optical positioning systems are limited by air turbulence and beam pointing stability. The beam intensity fluctuation and stochastic beam wandering were extensively studied and modeled in the case of laboratory and atmospheric conditions [12]. It is difficult to quote numbers for an accuracy as a function of the distance, since many factors are system-specific such as wavelength, beam diameter, temperature gradients, laminar or turbulent air flow, and scale of the vortices. Nevertheless, this beam wandering effect does not explain the poor results reported in the literature for straightness measurements over 1 cm to 1 m, which are precisely the distances of interest for an implementation in machine tools or measuring machines.

One generally assumes an ideal collimated Gaussian beam whose shape, or more precisely whose center of mass of the optical intensity distribution, does not vary over distance. In reality, the beam is not completely diffraction-limited, but is disturbed by small imperfections and reflections in the collimator lens assembly. Lenses are generally manufactured with an accuracy of λ/4; the aspheric lens design may not exactly be tailored to the laser wavelength, etc. All of these factors disturb the optical phase front, causing the beam intensity distribution to slightly vary with the distance, even with high-quality optical components. These variations in the beam are barely visible by the naked eye, which has a logarithmic sensitivity, and they are, therefore, not noticed by 4QD and PSD users. For typical beam diameters on the order of 1 mm to 5 mm, one can easily understand the best accuracy of 1 µm or worse reported in the literature for the position of the center of mass. However, one can overcome this limitation by realizing that this effect is configuration-specific, and it produces a systematic error, as long as the collimator is not re-adjusted. Systematic errors are easily eliminated by calibration using conventional error separation techniques [13]. This is what we report herein to drastically improve the accuracy of an optical straightness sensor. 

## 3. System Realization

The major component for all applications presented here is a monochrome CMOS image sensor with a digital interface and preferably a low power consumption. Image sensors are produced in a large variety, and many low-cost sensors are also available. In this work, we used a Basler monochrome image sensor model daA1280-54 um, which has a CMOS 1.2 megapixel chip (4.8 mm × 3.6 mm), a resolution of 1280 × 960 pixels, 3.75 µm square pixel size, a 12-bit digital-to-analog converter (DAC), a Universal Serial Bus (USB) 3 interface, and 1.2 W power consumption. As the image sensor has a high photosensitivity, it can be combined with a laser diode operated below the lasing threshold, delivering low-coherence light of less than 1 µW. The low coherence length suppresses interferences that originate from spurious reflections. Before any measurement, the image sensor sensitivity is optimized by adjusting gain and shutter speed to avoid saturation and to obtain the optimal signal-to-noise ratio. In our case, the gain was set to minimum and the shutter time to 0.43 ms for an optical beam intensity of 0.1 µW. Background light may disturb the measurement of the center of the beam; therefore, ambient light was reduced by appropriate means such as light traps for index sensors or lens tubes mounted in front of the straightness sensor. The algorithm used for finding the position of the beam center is based on a simple intensity-weighted mean. In order to reduce the sensitivity due to a possible inhomogeneous background illumination, an intensity threshold is implemented. More complex algorithms based on Gaussian fits were also tested but did not show much benefit with respect to the simple weighted mean method with a threshold. Most of our experiments were conducted with the room light switched off. In case this is too restrictive, modulating the laser diode and implementing a synchronous lock-in detection is a possible solution.

A major drawback of image sensors versus other types of optical sensors is their power dissipation, which can lead to thermal drift of the chip position, as stated in the results of our static test (Section 4.1). In our system, the image sensor is held by four screws maintaining its electronic board. A better solution would be to directly fix the CMOS image sensor chip. We did not implement this solution, but chose either to interrogate the image sensor at a constant rate in order to stabilize its temperature due to the stable continuous power consumption, or to switch off the power to the image sensor most of the time and power the sensor only during negligibly short interrogation periods.

### 3.1. Index Sensor and Position Sensor

**Figure 1:**
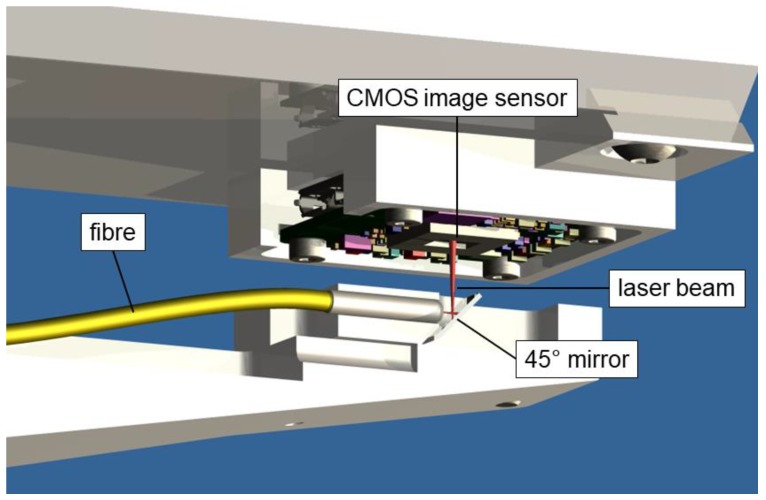
Configuration of the developed small-range two-dimensional (2D) position and index sensor.

**Figure 2:**
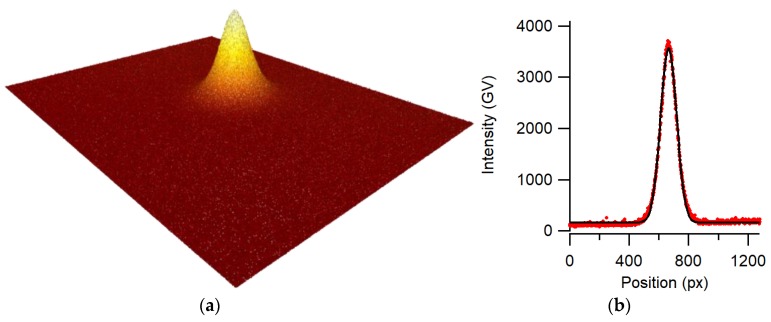
(**a**) Laser beam profile on the image sensor; (**b**) Gauss fit through its central profile.

For application as a two-dimensional (2D) index sensor, and also for direct small-range 2D position measurements, we used a light spot from an optical fiber end positioned close to the sensor surface. The bare fiber end shines directly onto the CMOS chip at a distance ranging between 1 mm and 5 mm. In order to build a compact system with a low height profile as shown in Figure 1, a 45° mirror reflecting the beam was added at the fiber end. Transmitting the light from the laser diode through a fiber considerably reduces the local thermal load near the image sensor and ensures an almost perfect spatial filtering, as shown in Figure 2.

### 3.2. Straightness Sensor

**Figure 3:**
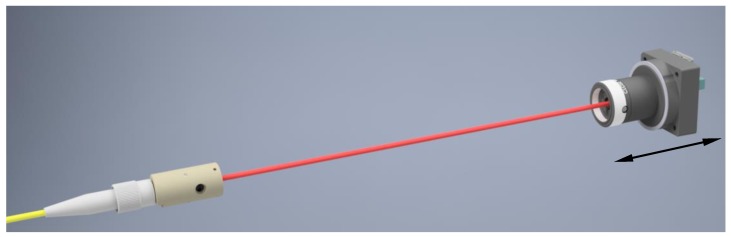
Configuration of the developed straightness sensor with an aspheric lens collimator.

For configuration as a straightness sensor, the fiber end was fitted with an aspheric lens collimator (focal length 10 mm) in order to form a Gaussian beam with a diameter of approximately 1 mm. With this configuration and an image sensor size of 4.8 mm × 3.6 mm, the working distances can easily reach up to 1.5 m. Figure 3 depicts the configuration of the used straightness sensor. The collimator is stationary, and the CMOS image sensor is attached to the stage carriage. The tube in front of the image sensors does not incorporate any lens; its only purpose is the reduction of stray light.

**Figure 4:**
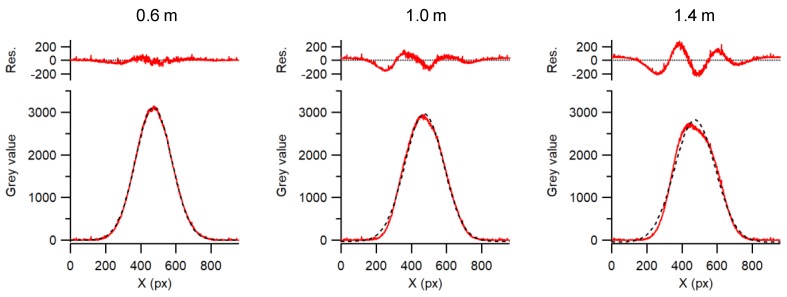
Central beam profiles at path lengths of 0.6 m, 1.0 m, and 1.4 m. The top plots (Res.) show the deviation between the profile and a Gaussian fit (dashed line).

Straightness measurements require a high-quality beam profile used as an optical reference line. Using the advantage of the image sensor, the beam quality can be measured online. Variations of the beam shape along the beam path can be detected and used as an indication of the quality of a straightness measurement. For this application, the laser beam was issued from a single-mode optical fiber pigtailed to a 670-nm laser diode. The single-mode fiber acts as a spatial filter, and we verified that higher-order modes were not present. The laser diode was driven below its lasing threshold, which reduced problems due to coherent reflections. For the collimator, a high-quality aspherical lens was used to try to avoid distortion of the wave front and reflections, as observed in multiple lens systems. Finding a suitable lens manufacturer was not easy, as we struggled with poor-quality lenses that distorted the beam shape and induced large systematic errors. Figure 4 shows beam profiles at different path lengths. It is evident that the beam profiles deviate from an ideal Gaussian shape, and their center of gravity is shifted, depending on the distance between the collimator and the image sensor. This effect is attributed to imperfectly collimated rays that cause spurious interferences on the image sensor. Using a good high-quality aspheric lens, the collimation of the beam could be precisely adjusted such that the intensity at the beam center and beam diameter viewed by the image sensor varied less than 1% over a 1-m travel range.

**Figure 5:**
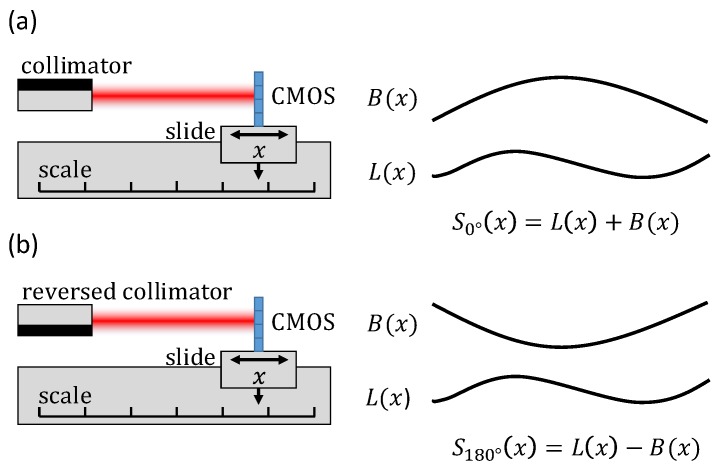
Schematic of the error separation technique that enables separating the optical system error *B(x)* from the linear stage straightness *L(x)*. Therefore, the indicated straightness *S_0°_(x)* and *S_180°_(x)* was measured with the collimator aligned 0° and 180° (reversed) relative to the beam axis.

To assess the performance of the straightness sensor, an error separation technique was applied [13]. As shown in Figure 5, it enables separating straightness deviations introduced by the linear stage *L(x)* from the optical system error *B(x)*, i.e., systematic errors issued from the beam shape. To this end, straightness deviations were recorded with the fiber and its collimator rotated at 0 and 180° around the beam axis. The indicated straightness *S(x)* for these measurements is given as follows:

(1) $$S_{0{}^{\circ}}\left(x\right)=L\left(x\right)+B\left(x\right),$$

(2) $$S_{180{}^{\circ}}\left(x\right)=L\left(x\right)-B\left(x\right),$$

where *x* is the position of the linear stage. By combining these equations, optical system errors *B(x)* were separated from the straightness of the displacement stage *L(x)* as follows:

(3) $$L\left(x\right)=\frac{S_{0{}^{\circ}}\left(x\right)+S_{180{}^{\circ}}\left(x\right)}{2},$$

(4) $$B\left(x\right)=\frac{S_{0{}^{\circ}}\left(x\right)-S_{180{}^{\circ}}\left(x\right)}{2}.$$

Assuming that *B(x)* is constant over time, it can be used to correct the stage straightness measurements from a look-up table using Equation (1).

## 4. Results and Discussion

### 4.1. Static Test Measurement

**Figure 6:**
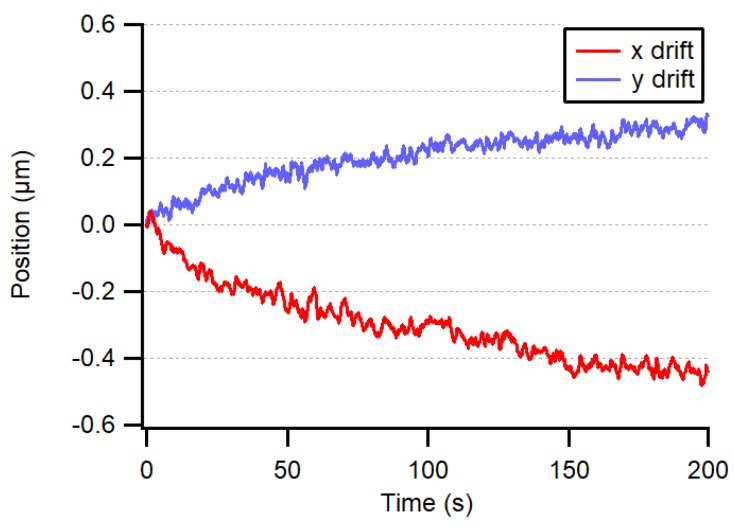
Drift of chip position as the system warms up from powered but idle at 0 fps to transmitting at 10 fps.

**Figure 7:**
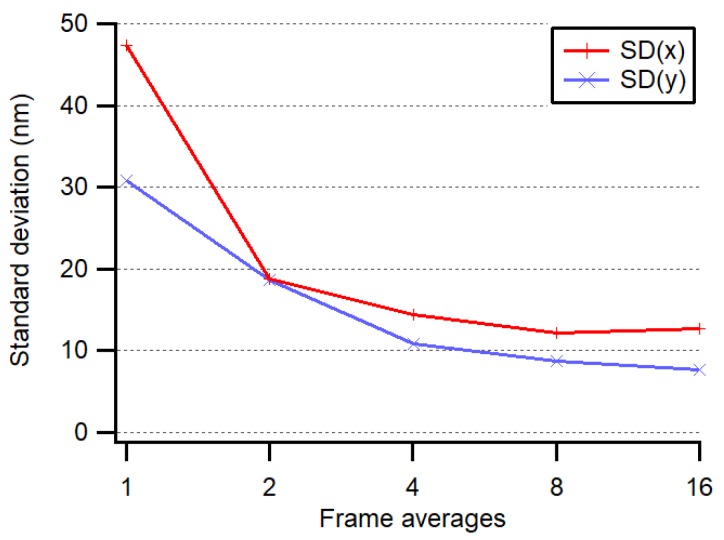
Averaging over several frames increases the accuracy; however, with more than four frames, little further benefit is observed.

In order to test the fundamental accuracy of our system, the bare fiber end was fixed directly to the image sensor housing, in the center, at about 2 mm from the CMOS chip surface. The threshold for eliminating background illumination was set to 6%. The center of the light spot was then computed to evaluate the fundamental accuracy of the system and the thermal drift of the sensor during warm-up. The drift, from powered but idle at 0 fps to transmitting at 10 fps, was less than 0.5 µm, as shown in Figure 6, thanks to the central position of the chip with respect to the image sensor mounting screws. To avoid this drift, the power of the image sensor was switched off most of the time except for during the negligibly short interrogation periods. The noise level of our measuring system was less than 15 nm (1 σ) when averaging four frames at 20 fps, as depicted in Figure 7.

### 4.2. Index Sensor and Position Sensor Measurements

**Figure 8:**
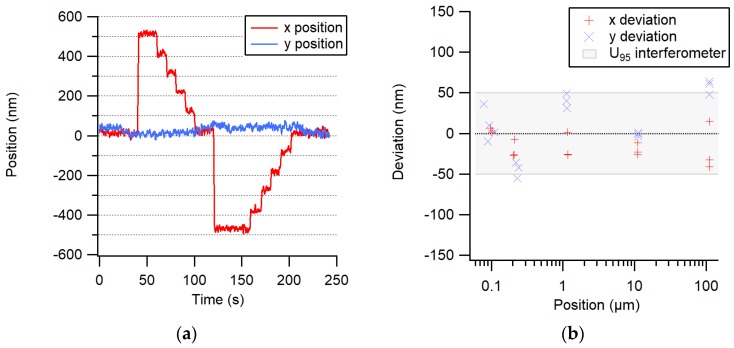
(**a**) The trajectory of 500 and 5 × 100 nm steps is clearly resolved. (**b**) Deviations of the image sensor measurements compared to an interferometer. The measurement uncertainty of the interferometer is indicated by the shaded area.

In a second experiment, the fiber end was mounted on a two-axis transverse displacement stage placed at a fixed 2-mm distance from the chip surface (Figure 1). The two-axis stage is part of the computed tomography system developed at METAS [9] and has a rather large metrology loop of about 1 m; however, it is situated in a thermally well-stabilized environment. The position of the stage was controlled in a closed loop using its own integrated line scale. A transverse trajectory of 500 nm steps up and 5 × 100 nm steps down, followed by a trajectory of 500 nm steps down and 5 × 100 nm steps up, was performed. Figure 8a shows the displacement of the center of the spot of light measured by the image sensor. One can notice that the 100-nm steps are clearly resolved. Next, the accuracy of larger displacements was evaluated by comparing them to an interferometric measurement, as shown in Figure 8b.

With this high resolution, this system is interesting for application as a 2D position sensor for small ranges or as an absolute index sensor for machines using an incremental displacement measuring system such as an interferometer.

### 4.3. Straightness Sensor Measurements

The straightness experiment configuration depicted in Figure 3 was realized with the distance between the collimator and image sensor ranging from 0.1 m to 1.5 m. Since the beam shape highly influences the straightness measurements, high-quality collimator lenses were used, and the beam profiles were characterized over the whole working range. To perform accurate straightness measurements, the error separation technique described in Section 3.2, which accounts for these deviations, is indispensable.

**Figure 9:**
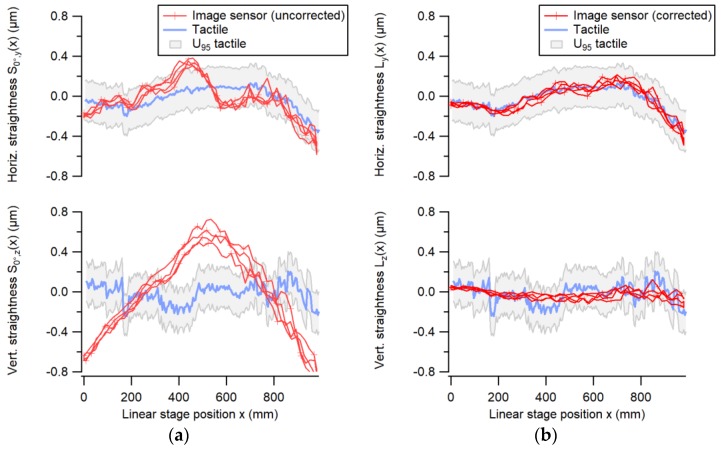
The uncorrected straightness indicated by the image sensor *S_0°_* (**a**), and the straightness of the linear stage after error separation *L* (**b**) over a range of 1000 mm. The straightness measured with the image sensors (red curves) is compared to tactile measurements obtained on a straight edge (blue curves).

The straightness measured by the image sensor was compared with the straightness of the displacement stage, which was calibrated using conventional methods, i.e., a tactile probe measuring a ceramic straight edge. Electronic levels were used to correct the angular errors introduced by the Abbe offset of the straight edge [14]. The measurement results shown in Figure 9 were determined over a range of 1000 mm along a horizontal linear axis. The optical system error induced by the non-ideal beam shape, changing over the travel range of the stage, was separated from the stage straightness by error separation. After correction, the two systems agreed within 100 nm for horizontal translational error motion and within 250 nm for vertical translational error motion. The larger deviations for the vertical straightness could be due to the specific air-bearing configuration of the employed linear stage.

**Figure 10:**
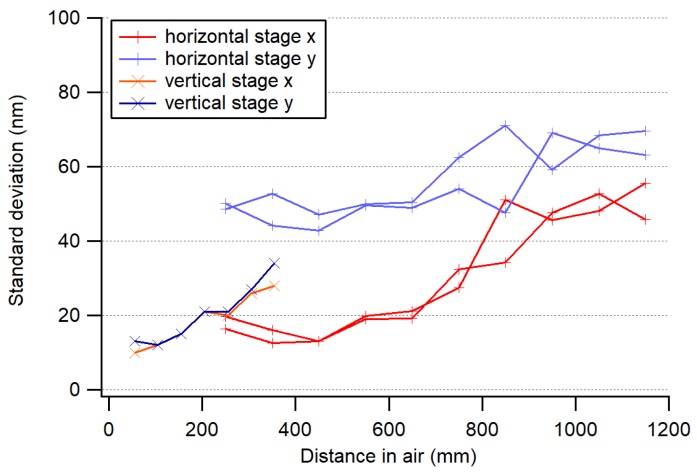
Evaluation of the straightness sensor noise (1 σ) over a range of 50 to 350 mm for a vertical linear axis and over a range of 200 to 1200 mm for a horizontal linear axis. Air-bearing fluctuations could be responsible for the 50-nm noise in the *y*-direction for the horizontal stage; *x* and *y* refer to the coordinate system of the image sensor.

The beam pointing stability was investigated at several distances, on a vertical axis with 300 mm range and a horizontal axis with 1000 mm range. Fluctuations increased with distance, as shown in Figure 10, which clearly indicates that they were induced by air turbulence of our climatized laboratory. The fluctuations of the air-bearing thickness also induced position fluctuations in one direction only. The standard deviation ranged from 15 nm at the 50-mm distance to about 70 nm at 1.2 m (bandwidth = 5 Hz).

### 4.4. Discussion

The static test exhibited an ultimate and stable resolution of 10 nm (1 σ), equal to a 300-fold sub-pixel interpolation. This could eventually be further enhanced since the system may be limited by the optical intensity fluctuations, the electronic noise of the image sensor, or the ambient light variations. Before further investigations, thermal drifts of the electronic board need to be eliminated by directly mounting the CMOS image sensor.

The test as an index and position sensor was clearly limited by thermal drifts and by the linear stage control loop; otherwise, its expected accuracy should be as good as shown in the static test.

The systematic errors in the optical beam shape, which are mainly caused by a non-ideal aspheric lens, were considered and corrected using the above-described error separation technique. The use of a CMOS imaging sensor helped us identify this error contribution as being a systematic and, thus, correctible error, which would go unnoticed when using a PSD or a 4QD. The magnitude of this error in our case was up to ± 0.8 µm, as seen in Figure 9, despite the use of very-high-quality optics. Once this systematic error was corrected, the accuracy of the straightness sensor configuration was mainly limited by air turbulence, which increased with distance and air-bearing noise. In order to mitigate this problem, one could measure several shorter straightness sections with lower noise and stitch them together to obtain the full-length straightness.

## 5. Conclusions

This study showed that our proposed simple 2D index and straightness measurement system can reach a very high accuracy at rather low cost. Once the warm-up and thermal fluctuations of the image sensor are mastered, the index sensor can clearly achieve an accuracy below 50 nm, whereas the straightness sensor achieved 100-nm accuracy over a 1-m travel range. An error separation method, by rotating the beam collimator by 180° around the propagation axis, was essential to reduce the systematic errors in the beam profile. Performances over even longer distances, up to several meters, are still under investigation. The proposed straightness measurement system is less costly and less cumbersome to use than conventional tactile or interferometric systems. It can be a potential alternative for machine calibration or even for an in situ real-time measuring system.
